# Developmental Origins of Chronic Kidney Disease: Should We Focus on Early Life?

**DOI:** 10.3390/ijms18020381

**Published:** 2017-02-11

**Authors:** You-Lin Tain, Chien-Ning Hsu

**Affiliations:** 1 Department of Pediatrics, Kaohsiung Chang Gung Memorial Hospital and Chang Gung University College of Medicine, Kaohsiung 833, Taiwan; tainyl@hotmail.com; 2 Institute for Translational Research in Biomedicine, Kaohsiung Chang Gung Memorial Hospital and Chang Gung University College of Medicine, Kaohsiung 833, Taiwan; 3 Department of Pharmacy, Kaohsiung Chang Gung Memorial Hospital, Kaohsiung 833, Taiwan; 4 School of Pharmacy, Kaohsiung Medical University, Kaohsiung 807, Taiwan

**Keywords:** chronic kidney disease, congenital anomalies of the kidney and urinary tract (CAKUT), developmental origins of health and disease (DOHaD), Epigenetic regulation, nephron endowment, oxidative stress, renin-angiotensin system, sex differences, sodium transporter, transcriptome

## Abstract

Chronic kidney disease (CKD) is becoming a global burden, despite recent advances in management. CKD can begin in early life by so-called “developmental programming” or “developmental origins of health and disease” (DOHaD). Early-life insults cause structural and functional changes in the developing kidney, which is called renal programming. Epidemiological and experimental evidence supports the proposition that early-life adverse events lead to renal programming and make subjects vulnerable to developing CKD and its comorbidities in later life. In addition to low nephron endowment, several mechanisms have been proposed for renal programming. The DOHaD concept opens a new window to offset the programming process in early life to prevent the development of adult kidney disease, namely reprogramming. Here, we review the key themes on the developmental origins of CKD. We have particularly focused on the following areas: evidence from human studies support fetal programming of kidney disease; insight from animal models of renal programming; hypothetical mechanisms of renal programming; alterations of renal transcriptome in response to early-life insults; and the application of reprogramming interventions to prevent the programming of kidney disease.

## 1. Introduction

**Figure 1:**
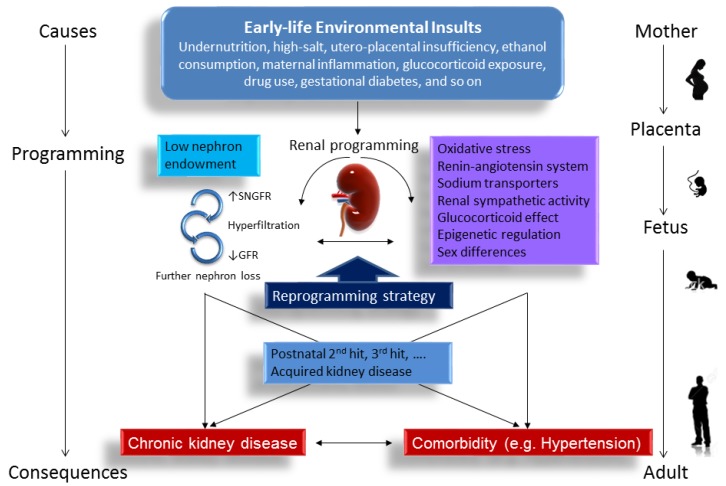
Schema outlining the early-life insults that induce renal programming and increase the vulnerability to later superimposed renal injury, leading to chronic kidney disease (CKD) and related comorbidity in later life. CKD can be attributed to multiple hits. A variety of early-life environmental insults (e.g., undernutrition) can cause renal programming, which is associated with low nephron endowment and other molecular mechanisms (e.g., oxidative stress). Renal programming likely constitutes a first-hit to the kidney which makes the kidney more vulnerable to postnatal insults (i.e., 2nd hit) to develop CKD in later life. The blue arrowhead indicates the application of reprogramming strategies in early life may prevent the developmental programming of kidney disease. SNGFR = single nephron glomerular filtration rate. GFR = glomerular filtration rate.

Chronic non-communicable diseases (NCDs) are the leading cause of mortality in the world and pose a great threat to human health [1]. NCDs can be driven by environmental insults in early life. This has been given rise to the concept of “developmental programming” or the “developmental origins of health and disease” (DOHaD) [2]. The term “developmental programming” refers to the process by which an insult applied at a critical window of development causes long-term effects on the structure or function of an organism [3]. Chronic kidney disease (CKD) has been recognized as a major NCD [4]. Despite recent advances in CKD management [5], there is still a global rising incidence and prevalence of CKD [4,6]. World Kidney Day 2016 focuses on childhood kidney disease and the antecedents of adult disease that can begin in early life [7]. Development of the kidney can be programmed in utero in response to an adverse condition. The human kidney does not start to function until after birth and its maturation continues for months, and up to years, after birth. Accordingly, the developing kidney is particularly vulnerable to adverse intrauterine and neonatal environments, causing permanent morphological changes and functional adaptation, that is, renal programming [8]. Although a variety of organ systems can be programmed in response to early-life insults, renal programming is considered key in the development of CKD and comorbid illness [9,10,11,12,13,14,15]. On the other hand, the DOHaD concept-based reprogramming strategies may provide a paradigm shift in therapeutic approaches from adulthood to early life, before CKD is evident [15]. A better understanding of the programming mechanisms in kidney development that lead to kidney disease is essential to developing early reprogramming intervention to halt the globally growing epidemic of CKD-related diseases. A schematic summarizing the links between early-life insults, mechanisms underlying renal programming, and programmed kidney disease is presented in Figure 1.

## 2. Evidence for Programming of Kidney Disease in the Human

Important support for the DOHaD concept came from observations on a cohort born during the Dutch Hunger Winter 1944–1945 [16], which demonstrated that malnutrition during gestation has long-lasting consequences for adult health. In the Dutch famine cohort, adults exposed to maternal famine had albuminuria [17], a diagnostic criteria of CKD associated with reduced nephron number [5]. A low nephron endowment is considered a common denominator underlying the susceptibility to kidney disease and comorbid illness [17,18]. In humans, nephrogenesis starts at gestational week 8–9 and ends at gestational week 36. Formation of a tree-like structure via the ramification of epithelial tubules during kidney development is called branching morphogenesis [19]. In the mammalian kidney, branching morphogenesis leads to the formation of the urinary collecting system and nephrons. Nephron endowment is largely determined through branching of the ureteric bud. Iterative branching of the ureteric bud occurs approximately 15 times during kidney development, generating an average ~1 million nephrons, but varies over a more than 10-fold range [20]. Thus, defective branching morphogenesis may lead to low nephron endowment and a broad range of clinical phenotypes, namely congenital anomalies of the kidney and urinary tract (CAKUT).

Full-term infants usually get a complete endowment of nephrons. However, preterm infants may develop low nephron endowment due to intra-uterine growth retardation (IUGR), compromised pregnancy, inadequacy of postnatal nutrition, and treatment with drugs, such as NSAIDs, after birth [14]. A nephron deficit can lead to higher glomerular capillary pressure and glomerular hyperfiltration, compensatory glomerular and tubular hypertrophy, consequently initiating a vicious cycle of further nephron loss in later life [20]. Since CKD can be attributed to multiple hits [21], a programmed low nephron endowment likely constitutes a first-hit to the kidney which makes the remaining glomeruli more susceptible to environmental influences and increases the vulnerability to develop CKD when facing renal injury in later life.

Numerous epidemiologic studies now support that low birth weight (LBW) and prematurity are risk factors for kidney disease in later life [11,14]; importantly, both are the robust clinical surrogates for low nephron endowment [17]. A meta-analysis of >2 million individuals reported that those with LBW had a 70% increased risk for development of CKD [22]. A case-control study of ~2000 children with CKD identified several prenatal and maternal factors, such as LBW, maternal gestational diabetes, and maternal obesity, impact the risk of CKD [23]. Additionally, our recent case-control study of >1.6 million infants found that risk factors for CAKUT include prematurity, LBW, maternal gestational diabetes, maternal thalassemia/hemochromatosis, polyhydramnios or oligohydramnios, male, and first parity [24]. Unlike adults, CAKUT is a major cause of CKD in children [7]. CAKUTs contain a wide range of renal system structural malformations, characterized by varying deficits in nephron number [25]. The low nephron endowment observed in CAKUT may attribute to the prevalence of CKD in individuals with CAKUT.

Unfortunately, the number of nephrons cannot be determined in vivo thus far. Although the use of ferritin-based nanoparticles as targeted magnetic resonance imaging (MRI) contrast agent to measure nephron number in human kidneys has made some progress [26], the validation of non-invasive method to evaluate nephron endowment in vivo deserve greater efforts. To date, the nephron endowment can only be estimated via the surrogate markers. As reviewed elsewhere [17,18], clinical surrogates for low nephron endowment include LBW, prematurity, short stature, low kidney mass and volume, gene polymorphisms, maternal gestational hyperglycemia, and gender. Although numerous nutritional interventions have been effective in reducing the risk of LBW and prematurity [14], their impacts on nephron endowment and programming of adult kidney disease remain unclear.

A number of hypotheses, such as thrifty phenotype [27], predictive adaptive responses [28], and catch-up growth hypothesis [29], have been developed to explain the epidemiological observations of an association between early life insults and later chronic diseases. However, these hypotheses do not suggest molecular mechanisms whereby the phenotype is generated. Despite the risk of kidney disease has been assessed in a number of human studies, interventions necessary to prove causation and to provide a reprogramming strategy remain unaware. It is for this reason that much of our knowledge of the types of insults driving renal programming, the critical window of vulnerability for insults, potential mechanisms of renal programming, and reprogramming strategy come from studies in animal models.

## 3. Animal Models of Renal Programming

A number of animal studies confirm the association between early-life insults, renal programming, and subsequent CKD in adulthood. Here we summarize studies previously reviewed [9,10,15,18] and highlight new data documenting renal programming associated with low nephron number (Table 1). Renal development in rodents, unlike in humans, continues up to postnatal week 1–2. Therefore, adverse conditions during pregnancy and early lactation period may impair nephrogenesis, leading to renal programming and adult kidney disease. As shown in Table 1, a variety of pre-, peri-, and post-natal insults have been reported to cause renal programming and low nephron endowment. These factors include maternal undernutrition, high-salt, low-salt, utero-placental insufficiency, ethanol consumption, maternal inflammation, glucocorticoid exposure, vitamin A deficiency, iron deficiency, drug use, and gestational diabetes [30,31,32,33,34,35,36,37,38,39,40,41,42,43,44,45]. 

Reduced nephron number can develop from birth through adulthood to old age in different experimental models of renal programming. Insults need only last for a brief moment during nephrogenesis, as little as 1–2 days, to cause a permanent reduced nephron endowment [34,37]. In rats, maternal administration of dexamethasone for 2 days results in a reduced nephron number in adult offspring when administered on embryonic day 13–14 or 17–18 [37]. These finding indicate there is a critical window of vulnerability for insult during kidney development. The main phenotype of renal programming associated with low nephron endowment is glomerular hypertrophy. A reduction of nephron number, in the absence of compensatory hypertrophy, would be expected to cause a decreased glomerular filtration rate (GFR). As shown in Table 1, however, variations of GFR observed in different models of renal programming can be reduced [33,34,35,36], unaltered [37,39,40,41,44], or even augmented [42]. These data suggest that there is a differential degree of compensatory hypertrophy in the setting of a low nephron endowment in various models of renal programming.

On the other hand, nephron endowment can be unaltered [46], or even increased in response to renal programming [47,48]. Thus, low nephron endowment, per se, does not appear to mediate all programmed processes related to the development of kidney disease. These findings implicate that the renal programming is not specific to a single factor (i.e., low nephron endowment) and other mechanisms of renal programming demands further analysis. 

## 4. Mechanisms of Renal Programming

So far, a number of hypothetical mechanisms, including oxidative stress, alterations of renin-angiotensin system (RAS) and sodium transporters, renal sympathetic activity, glucocorticoid effect, epigenetic regulation, and sex differences have been reported to be associated with altered development of kidney structure or function, that is, renal programming [8,9,10,11,12,13,14,15]. Each will be discussed in turn. 

### 4.1. Oxidatice Stress

Oxidative stress, arising as a result of an imbalance between free radical production (e.g., reactive oxygen species (ROS)) and antioxidant defenses. The normal redox status plays a key role in fetal development. The development of embryo occurs in a relatively low-oxygen environment [49]. It is highly vulnerable to oxidant injury. A number of animal models suggest oxidative stress involved in renal programming, including caloric restriction [40], maternal diabetes [41], prenatal dexamethasone exposure [43], high fructose intake [50], prenatal dexamethasone and postnatal high-fat diet [51], preeclampsia [52,53], maternal smoking [54], and low-protein diet [55]. Additionally, emerging evidence supports that NO-ROS imbalance is important for programmed hypertension [15,56]. Despite current advances in understanding of how early-life redox imbalance impacts renal programming, further studies are needed to establish the particular developmental window and kidney-specific redox-sensitive signaling responsible for these redox changes [57].

### 4.2. Renin-Angiotensin System

The RAS is critical in mediating proper nephrogenesis and regulating renal physiology [58,59]. This system is comprised of different angiotensin peptides with diverse biological functions mediated by distinct receptors. The classic RAS, defined as the angiotensin converting enzyme (ACE)-angiotensin (Ang) II-angiotensin type 1 receptor (AT1R) axis, promotes vasoconstriction and sodium retention. In contrast, the non-classical RAS composed of the ACE2-Ang-(1–7)-MAS receptor is a new opposing axis, leading to vasodilatation [59]. Although both axes of the RAS have been examined on their roles in fetal programming [60,61], studies to date have produced conflicting results with up- and down-regulation of almost all components of the intrarenal RAS being reported [8]. There is a biphasic response with reduced RAS expression at birth that becomes normalized with age. However, this normalization in the adult may be inappropriately high and, hence, activating the classical RAS during kidney development [8]. So far, there are a few studies showing that blockade of the classical RAS in early life between 2–4 weeks of age can offset the effects of developmental programming [62,63,64,65,66], supporting targeting on the RAS might be a reprogramming strategy to prevent programmed kidney disease. Nevertheless, there remains a lack of definitive data on how and when to target components of the RAS to prevent the programming of kidney disease and its related comorbidities. 

### 4.3. Sodium Transporters

Renal reabsorption of sodium is a part of renal physiology. Renal transporters have been examined in different models of renal programming, especially prenatal glucocorticoid exposure [36,37,43] and low-protein diet [38,67]. These studies have demonstrated that prenatal insult-induced renal programming is related to the increases of renal mRNA levels, protein abundance, and/or activity of several sodium transporters including Na-K-2Cl cotransporter (NKCC2), type 3 sodium hydrogen exchanger (NHE3), Na^+^/Cl^−^ cotransporter (NCC), and Na^+^/K^+^ATPase α1 subunit (NaKATPase). Similarly, we observed that maternal high-fructose diet plus postnatal high-salt diet increased renal levels of NKCC2, NHE3, and NCC in a two-hit model of renal programming [68]. Thus, renal programming models, no matter whether there was prenatal or postnatal exposure, may elicit inappropriate sodium reabsorption that increase the vulnerability to develop adult kidney disease. It is noteworthy that serum- and glucocorticoid-inducible kinase (SGK1) contributes to the regulation of almost all sodium transports, which can be activated by glucocorticoid signaling, as well as by salt [69,70]. These data suggest that SGK1 may be a reprogramming target for the programmed kidney disease.

### 4.4. Renal Sympathetic Activity

In the rat, both afferent and efferent nerves are observed inside the developing kidney by embryonic day 16 [71]. The nerves start to enter the kidney at late gestation, reach the outer cortical renal arterioles at postnatal week 1–2, and continue to mature into postnatal life [72]. Hence, a suboptimal environment during pregnancy and early lactation period may impact renal sympathetic activity, as reported in a number of renal programming models [67,73,74]. On the other hand, renal denervation can attenuate programmed hypertension in renal programming models with placental insufficiency [74] and prenatal dexamethasone exposure [67]. In addition, renal sympathetic nerve activity plays a crucial role in renin secretion and sodium reabsorption. Since the RAS cascade starts with the release of renin, and because the RAS and sodium transporters are both important mechanisms involved in renal programming, the interactions between renal sympathetic activity, RAS, and sodium transporters leading to adult kidney disease awaits further elucidation. Our recent study demonstrated that prenatal dexamethasone exposure increased renin (fold change = 2.41) and (pro)renin receptor (fold change = 2.37) mRNA expression during nephrogenesis [75]. Additionally, we found that maternal high-fructose-induced renal renin expression from one day (fold change = 3.05) to three months (fold change = 3.38) of age [76]. These findings imply that renal sympathetic activity and its downstream signaling may contribute to renal programming and kidney disease in later life. 

### 4.5. Glucocorticoid Effect

Glucocorticoids are critical in normal development and organogenesis of the fetus. In normal pregnancy, glucocorticoid levels are much lower in the fetal circulation compared to maternal circulation at term [77]. The fetus is protected by the placental inactivation of active glucocorticoids via the enzyme 11β-hydroxysteroid dehydrogenase type 2 (11β-HSD2). A variety of adverse conditions in utero, such as maternal malnutrition [78], maternal stress [79], and preeclampsia [80] have been reported to inhibit 11β-HSD2, resulting in fetal exposure to excess glucocorticoid. Although the effects of glucocorticoid-mediated programming have been examined in several systems and diseases [77,81,82,83], few studies have investigated the effects of glucocorticoids on the developing kidney. We recently utilized RNA next-generation sequencing (NGS) to analyze the renal transcriptome in the rat offspring at one and 16 weeks of age, to examine whether key genes and pathways are responsible for prenatal dexamethasone-induced renal programming [75]. Prenatal dexamethasone exposure results in alterations of 431 renal transcripts in the offspring at one and 16 weeks of age in a consistent manner. We analyzed a panel of genes that has previously been reported to be relevant to kidney development [84]. Among them, *Gfra1* (encodes for glial cell line-derived neurotrophic factor (GDNF) family receptor α 1) and *Cdh6* (encodes for cadherin 6, a membrane protein) were shared by one and 16 weeks of age. *Gfra1*, which is a member of the GDNF/Ret signaling controls branching morphogenesis. Knockout of *Gfra1* can cause the failure of ureteric bud outgrowth, leading to renal agenesis [85]. Next, *Cdh6* mediates cell–cell binding. The roles of *Gfra1* and *Cdh6* in the renal programming and low nephron endowment await further elucidation.

### 4.6. Epigenetic Regulation

Epigenetics refers to alterations in gene expression that are not explained by changes in the DNA sequence. Unlike genetic information, epigenetic events are reversible and respond to environmental insults. The three major epigenetic factors are as follows: DNA methylation, histone modification, and microRNA (miRNA)-mediated silencing [86]. Global methylation patterns have been studied in several programming models, such as maternal low-protein diet [87], maternal tobacco use [88], and micronutrient deficiency [89]. However, little attention has been paid to the kidney, except one recent study showed that maternal folic acid supplementation did not alter global DNA methylation in offspring kidney [90]. Next, histone acetylation is one of the most frequent epigenetic modifications. Histone acetyltransferases (HATs) and histone deacetylases (HDACs) determine histone acetylation and deacetylation, respectively. Our previous work demonstrated that HDAC inhibition by trichostatin A prevented dexamethasone-induced programmed hypertension in a rat model of neonatal dexamethasone exposure [91]. Given that HDAC inhibitors have been proposed to test the therapeutic effects in the pre-clinical model of CKD [92], further studies are warranted to examine their reprogramming effects on the developmental programming of CKD. Aside from DNA methylation and histone modification, miRNAs may also play a key role in the fetal programming [93]. Recent microarray studies demonstrated that maternal nutrient restriction can permanently alter the expression of a variety of miRNAs in the aortas of rat offspring [94]. Another study showed renal miRNA modulation in the protein restriction model of fetal programming [95]. Moreover, circulating hypoxia-regulated miRNAs were increased in pregnant women with fetal growth restriction [96]. The roles of DNA methylation, histone modification, and miRNAs altering the expression of genes involved in the renal programming remain to be identified, but are the subject of great interest.

### 4.7. Sex Differences

There is increasing evidence that sex differences exist in the fetal programming of kidney disease and hypertension [97,98], showing that males are more vulnerable than females. In fact, several hypothetical mechanisms of renal programming, such as the RAS [99] and oxidative stress [100], have been reported to respond to environmental stress in a sex-specific manner. Additionally, the renal transcriptome is sex-specific [101,102]. In a prenatal dexamethasone exposure model [102], we found prenatal dexamethasone exposure induced sex-specific increases in blood pressure in male, but not female, adult offspring. We also observed sex-specific expression of *Agt*, *Agtr1a*, and *Agtr2* in the RAS, which was not altered by dexamethasone exposure. Our data suggest that the resistance of female offspring to prenatal dexamethasone-induced programmed hypertension is related to a lower Agt mRNA expression. Furthermore, another line of evidence supports the impact of sex differences on renal programming; namely, for maternal high-fructose intake altered renal transcriptome of both sexes at one week of age, female offspring are more fructose-sensitive [66]. This is in accord with literature documenting that more genes in the placenta were affected in females than in males under different maternal diets [103,104]. However, whether the increased female sensitivity to insults is beneficial or harmful for programming of female fetuses remains unclear. Thus, better understanding of the sex-dependent mechanisms that underlie renal programming will help develop a novel sex-specific strategy to prevent programmed kidney disease and comorbid illness in both sexes.

However, there is still lack of animal studies addressing multiple mechanisms simultaneously to explore their interrelationship and relative importance in different models of renal programming. Investigation of a wide spectrum of mechanisms and assessment of reprogramming therapies in animal models before applying their discoveries to humans is still a faraway goal.

## 5. Changes in Renal Transcriptome in Response to Early-Life Insults

Although several hypothetical mechanisms discussed above have been proposed to explain renal programming in diverse programming models, none of them are able to define the common genes and pathways that drive the programmed process. So far, only a few genome-wide studies have been conducted to identify the changes of renal transcriptome exposed to different early-life insults [105,106,107,108,109]. Since nephrogenesis completes at postnatal week 1–2 in rodents, analyzing the renal transcriptome right after the completion of nephrogenesis might aid in identifying the primary programmed changes in response to environmental insults. Therefore, we used NGS techniques to quantify the abundance of RNA transcripts in two-week-old offspring kidneys that had maternal exposure to caloric restriction, STZ-induced diabetes, high-fructose consumption, and high salt intake [109]. We identified 809, 965, 356, and 272 differentially expressed genes (DEGs) in the models of caloric restriction, diabetes, high-fructose, and high salt, respectively. Although a total of 16 DEGs were shared among four different models, to the best of our knowledge, none of them have shown a direct relationship with programming of kidney disease. 

Additionally, we found a number of significantly related Kyoto Encyclopedia of Genes and Genomes (KEGG) pathways in the offspring kidneys (Table 2). Among them, the peroxisome proliferator-activated receptor (PPAR) signaling pathway and the glutathione metabolism pathway were shared by three different programming models. Even though the PPARs have been implicated in kidney disease and hypertension [110,111], there remains a lack of definitive data on how and when to prevent the developmental programming of kidney disease via targeting on PPARs in early life. Glutathione (GSH) is the major intracellular antioxidant [112]. Our previous study demonstrated that *N*-acetylcysteine can increase GSH and reduce oxidative stress to prevent the development of hypertension in different models of renal programming [51,52,53]. Additional studies are required to unravel the impacts of the glutathione pathway on oxidative stress and programmed kidney disease.

Notably, the arachidonic acid metabolism pathway is involved in maternal high-fructose intake-induced renal programming. Our recent studies indicate that early postnatal treatment targeting the arachidonic acid metabolism pathway by using a soluble epoxide hydrolase (SEH) inhibitor 12-(3-adamantan-1-yl-ureido)-dodecanoic acid (AUDA) ameliorates programmed hypertension in both programming models of maternal high-fructose consumption and prenatal dexamethasone exposure [113,114]. It would be interesting to understand whether SEH inhibition also prevents programmed kidney disease in other models of programming.

Furthermore, we analyzed a panel of genes relevant to kidney development [84,106]. As shown in Table 3, a total of four, 12, three, and two DEGs involved in nephrogenesis were identified in the models of caloric restriction, diabetes, high-fructose consumption, and high salt intake, respectively. Among them, *Gdnf* was commonly shared by the four different models. The *Gdnf* gene encodes for glial cell line-derived neurotrophic factor (GDNF), which is required for the morphogenesis of the ureteric bud during kidney development [115]. Additionally, we observed that *Fgf2* and *Osr1* were shared by caloric restriction and maternal diabetes, while *Six1* was shared by diabetes and high-fructose intake. Whether these genes are commonly related to low nephron endowment in response to different prenatal insults awaits further evaluation.

Our data indicate that a diverse range of early-life insults can generate differentially programmed processes. Since we only analyzed one time point at the completion of nephrogenesis in this study, further studies are warranted to examine the renal transcriptome at different developmental windows to provide us the whole picture of sequential changes of renal programming in response to early-life insults. 

## 6. Reprogramming Strategy to Prevent the Programming of Kidney Disease

With an understanding of the effects that renal programming have on the development of adult kidney disease, the invention of reprogramming strategy is a key priority. Reprogramming strategies need to be directed toward the types of early-life insults or mechanisms of renal programming. Early interventions to offset the programming of kidney disease might include a balanced diet for pregnant women with nutritional excesses or deficiencies, identification of individuals at risk of kidney disease based upon early screening of maternal and birth characteristics, or therapeutic approaches during early childhood to target gatekeeper or related programming processes. 

There are already examples of these kinds of reprogramming interventions in animal models. Several pathogenic mechanisms involved in renal programming, as we discussed above, can be the target for early intervention. Previous studies indicate that early treatment with α-tocopherol [116], quabain (an inhibitor of NaKATPase) [117], or retinoid acid [118] ameliorates reductions in nephron endowment in different programming models. We recently reviewed a numbers of reprogramming interventions that have been directed at restoring the balance of NO and ROS [15]. These interventions, including arginine, citrulline, vitamin C, vitamin E, melatonin, and lazaroid (an inhibitor of lipid peroxidation) have been used to prevent the oxidative stress-related adverse effects in a variety of programming models. Although ACE inhibitors and angiotensin receptor blockers (ARBs) are contraindicated during pregnancy, blockade of RAS in young offspring from ages 2 to 4 weeks can be preventive against the fetal programming of hypertension in animal models of programming [62,63,64,65,66]. These protective effects are not only directed upon the RAS, but also through regulating sodium transporters. As we mentioned earlier, renal denervation is effective to target renal sympathetic activity as a reprogramming therapy in both programming models of placental insufficiency and prenatal dexamethasone exposure [67,74]. Importantly, even these mechanisms are not primary causal factors, and these reprogramming interventions seem applicable to a wide spectrum of programming models. 

## 7. Conclusions

The global burden of CKD continues to increase, despite treatment advances [4,5,6]. CKD affects millions of people all over the world, including many children who are at risk at an early age. In 2016, World Kidney Day is dedicated to kidney disease in childhood [7]. The opportunity is here; should we be starting earlier? Major progress has been made in research on renal programming in animal studies, but many challenges still lie ahead. This review has summarized major mechanisms relevant to renal programming published in the literature, but it is still not complete. What is missing from the literature is a greater understanding of how renal programming interacts with extrarenal factors and responds to challenges in later life to induce CKD. A deeper understanding of the critical window for reprogramming interventions is also required. Future programming research should aim at filling the translational gap between animal models and clinical trials. Research into the preventions and treatments of CKD that begin early in life will have a lifelong impact and profound savings in disease burden and financial costs. 

## Figures and Tables

**Table 1:** Effects of early-life insults on renal programming with low nephron number in animal models.

Experimental Model	Renal Phenotype	Age at Evaluation of Nephron Endowment	Ref.
Uteroplacental insufficiency	↑ Apoptosis	1 day	[30]
Vitamin A-deficient diet from 3 weeks before mating throughout pregnancy	Not evaluated	1 day	[31]
Low sodium diet (0.07%) during pregnancy and lactation	Hypertension at 5 months	1 week	[32]
High sodium diet (3%) during pregnancy and lactation	Glomerular hypertrophy, hypertension at 5 month	1 week	[32]
Partial ligation of uterine ligation	↓ GFR, glomerular hypertrophy	2 weeks	[33]
Ethanol (1 g/kg/day) at gestational day 13.5 and 14.5	↓ GFR at 6 months	4 weeks	[34]
Lipopolysaccharide (0.79 mg/kg/day) i.p. at gestational day 8, 10, and 12	↓ GFR	7 weeks	[35]
Dexamethasone (0.1 mg/kg/day) throughout pregnancy	↓ GFR, glomerular hypertrophy	2 months	[36]
Dexamethasone (0.2 mg/kg/day) at gestational day 15 and 16 or 17 and 18	↔ GFR, unchanged glomerular morphology	2 months	[37]
Low protein diet (8% protein) during lactation	Hypertension at 5 months	2 months	[38]
Cyclosporine (3.3 mg/kg/day) from gestational day 10 to postnatal day 7	↔ GFR, glomerular hypertrophy	3 months	[39]
50% caloric restriction during pregnancy and lactation	↔ GFR, glomerular hypertrophy, hypertension, tubulointerstitial injury	3 months	[40]
Streptozotocin (STZ)-induced diabetes during pregnancy	↔ GFR, hypertension, tuburointerstitial injury	3 months	[41]
Multideficient diet during pregnancy	↑ GFR, glomerular hypertrophy	3 months	[42]
Dexamethasone (0.1 mg/kg/day) from gestational day 16 to 22.	Hypertension	4 months	[43]
Low protein diet (8.5% protein) during pregnancy	↔ GFR, hypertension	5.5 months	[44]
Iron restriction diet (3 mg/kg diet) from 1 week before mating and through pregnancy	Glomerular hypertrophy, hypertension	18 months	[45]

Studies tabulated according to age at evaluation. GFR = glomerular filtration rate. ↑ = increased. ↓ = decreased. ↔ = unaltered.

**Table 2:** Significantly regulated Kyoto Encyclopedia of Genes and Genomes (KEGG) pathways in the two-week-old offspring kidneys of maternal caloric restriction, diabetes, high-fructose, and high salt intake.

Caloric Restriction	Diabetes
Ribosome	Ribosome
Cell cycle	ABC transporters
Oocyte meiosis	Complement and coagulation cascades
DNA replication	Spliceosome
Fatty acid metabolism	Antigen processing and presentation
Tryptophan metabolism	Prostate cancer
Homologous recombination	Drug metabolism
Progesterone-mediated oocyte maturation	Histidine metabolism
Valine, leucine, and isoleucine degradation	Metabolism of xenobiotics by cytochrome P450
Prostate cancer	ECM-receptor interaction
PPAR signaling pathway	Tryptophan metabolism
Glutathione metabolism	Glutathione metabolism
Arginine and proline metabolism	PPAR signaling pathway
High fructose	High salt
PPAR signaling pathway	Cell adhesion molecules (CAMs)
Butanoate metabolism	Complement and coagulation cascades
Arachidonic acid metabolism	Hematopoietic cell lineage
Fatty acid metabolism	Systemic lupus erythematosus
Glutathione metabolism	Intestinal immune network for IgA production
Metabolism of xenobiotics by cytochrome P450	Graft-versus-host disease
Tyrosine metabolism	Allograft rejection
Drug metabolism	

**Table 3:** Fold changes in significantly differentially expressed genes involved in kidney development in the kidneys of offspring at two weeks of age exposed to maternal caloric restriction (CR), streptozotocin (STZ)-induced diabetes, high-fructose (HF) diet, and high salt (HS) intake.

Gene ID	Gene Symbol	CR	STZ	HF	HS
Expansion and survival of renal stem cells					
ENSRNOG00000012278	*Fgf10*	0.52	**0.38**	0.55	0.87
Formation and extension of the primary nephric duct					
ENSRNOG00000012819	*Gdnf*	**2563**	**1508**	**1836**	**1362**
ENSRNOG00000008430	*Spry3*	ND	**123**	1.04	**278**
ENSRNOG00000022777	*Six1*	1.58	**0.4**	**2.64**	1.55
ENSRNOG00000026053	*Grem1*	0.57	**0.47**	1.21	0.83
Initiation of metanephric development					
ENSRNOG00000003807	*Wnt9b*	0.75	1.27	**0.49**	0.85
ENSRNOG00000015982	*Wnt11*	1.18	**3.37**	1.29	1.3
ENSRNOG00000007002	*Lif*	**0.36**	0.64	0.95	1.12
ENSRNOG00000017392	*Fgf2*	**2.07**	2.9	1.54	0.82
ENSRNOG00000020792	*Etv4*	0.94	2.66	1.7	1.53
Mesoderm patterning					
ENSRNOG00000004210	*Osr1*	**0.27**	**0.46**	0.61	0.57
ENSRNOG00000021276	*Bmp2*	1.72	**2.39**	0.86	1.05
ENSRNOG00000000556	*Nodal*	ND	ND	ND	**242**
Nephron development					
ENSRNOG00000004517	*Igf1*	0.55	**0.44**	0.77	0.64
ENSRNOG00000004346	*Notch3*	1.18	**2.09**	0.83	1.06

ND = not detectable; Significant results are highlighted in bold.

## Abbreviations

ACE	Angiotensin converting enzyme
ARB	Angiotensin receptor blocker
CAKUT	Congenital anomalies of the kidney and urinary tract
CKD	Chronic Kidney Disease
DEG	Differentially expressed gene
DOHaD	Directory of open access journals
GSH	Glutathione
HDAC	Histone deacetylases
KEGG	Kyoto Encyclopedia of Genes and Genomes
LBW	Low birth weight
miRNA	MicroRNA
NaKATPase	Na^+^/K^+^ATPase α1 subunit
NCD	Non-communicable disease
NCC	Na^+^/Cl^−^ cotransporter
NGS	Next-generation sequencing
NHE3	Type 3 sodium hydrogen exchanger
NKCC2	Na-K-2Cl cotransporter
PPAR	Peroxisome proliferator-activated receptor
RAS	Renin-angiotensin system
ROS	Reactive oxygen species
SEH	Soluble epoxide hydrolase
STZ	Streptozotocin
11β-HSD2	11β-hydroxysteroid dehydrogenase type 2

## References

[1] Zarocostas J. (2010). Need to increase focus on non-communicable diseases in global health, says WHO. Br. Med. J..

[2] Hanson M., Gluckman P. (2011). Developmental origins of noncommunicable disease: Population and public health implications. Am. J. Clin. Nutr..

[3] Lucas A. (1998). Programming by early nutrition: An experimental approach. J. Nutr..

[4] Couser W.G., Remuzzi G., Mendis S., Tonelli M. (2011). The contribution of chronic kidney disease to the global burden of major noncommunicable diseases. Kidney Int..

[5] Kidney Disease: Improving Global Outcomes (KDIGO) CKD Work Group (2013). KDIGO 2012 clinical practice guideline for the evaluation and management of Chronic Kidney Disease. Kidney Int. Suppl..

[6] National Institutes of Health, National Institute of Diabetes and Digestive and Kidney Diseases (2013). U.S. Renal Data System, USRDS 2013. Annual Data Report: Atlas of Chronic Kidney Disease and End-Stage Renal Disease in the United States.

[7] Ingelfinger J.R., Kalantar-Zadeh K., Schaefer F., World Kidney Day Steering Committee (2016). World Kidney Day 2016: Averting the legacy of kidney disease-focus on childhood. Pediatr. Nephrol..

[8] Kett M.M., Denton K.M. (2011). Renal programming: Cause for concern?. Am. J. Physiol. Regul. Integr. Comp. Physiol..

[9] Chong E., Yosypiv I.V. (2012). Developmental programming of hypertension and kidney disease. Int. J. Nephrol..

[10] Paixão A.D., Alexander B.T. (2013). How the kidney is impacted by the perinatal maternal environment to develop hypertension. Biol. Reprod..

[11] Luyckx V.A., Bertram J.F., Brenner B.M., Fall C., Hoy W.E., Ozanne S.E., Vikse B.E. (2013). Effect of fetal and child health on kidney development and long-term risk of hypertension and kidney disease. Lancet.

[12] Boubred F., Saint-Faust M., Buffat C., Ligi I., Grandvuillemin I., Simeoni U. (2013). Developmental origins of chronic renal disease: An integrative hypothesis. Int. J. Nephrol..

[13] Singh R.R., Denton K.M. (2015). Role of the kidney in the fetal programming of adult cardiovascular disease: An update. Curr. Opin. Pharmacol..

[14] Luyckx V.A., Brenner B.M. (2015). Birth weight, malnutrition and kidney-associated outcomes—A global concern. Nat. Rev. Nephrol..

[15] Tain Y.L., Joles J.A. (2015). Reprogramming: A preventive strategy in hypertension focusing on the kidney. Int. J. Mol. Sci..

[16] Roseboom T., de Rooij S., Painter R. (2006). The Dutch famine and its long-term consequences for adult health. Early Hum. Dev..

[17] Painter R.C., Roseboom T.J., van Montfrans G.A., Bossuyt P.M., Krediet R.T., Osmond C., Barker D.J., Bleker O.P. (2005). Microalbuminuria in adults after prenatal exposure to the Dutch famine. J. Am. Soc. Nephrol..

[18] Luyckx V.A., Brenner B.M. (2010). The clinical importance of nephron mass. J. Am. Soc. Nephrol..

[19] Shah M.M., Sampogna R.V., Sakurai H., Bush K.T., Nigam S.K. (2004). Branching morphogenesis and kidney disease. Development.

[20] Bertram J.F., Douglas-Denton R.N., Diouf B., Hughson M.D., Hoy W.E. (2011). Human nephron number: Implications for health and disease. Pediatr. Nephrol..

[21] Brenner B.M., Garcia D.L., Anderson S. (1988). Glomeruli and blood pressure. Less of one, more the other?. Am. J. Hypertens..

[22] Nenov V.D., Taal M.W., Sakharova O.V., Brenner B.M. (2000). Multi-hit nature of chronic renal disease. Curr. Opin. Nephrol. Hypertens..

[23] White S.L., Perkovic V., Cass A., Chang C.L., Poulter N.R., Spector T., Haysom L., Craig J.C., Salmi I.A., Chadban S.J. (2009). Is low birth weight an antecedent of CKD in later life? A systematic review of observational studies. Am. J. Kidney Dis..

[24] Hsu C.W., Yamamoto K.T., Henry R.K., de Roos A.J., Flynn J.T. (2014). Prenatal risk factors for childhood CKD. J. Am. Soc. Nephrol..

[25] Tain Y.L., Luh H., Lin C.Y., Hsu C.N. (2016). Incidence and risks of congenital anomalies of kidney and urinary tract in newborns: A population-based case-control study in Taiwan. Medicine.

[26] Beeman S.C., Cullen-McEwen L.A., Puelles G., Zhang M., Wu T., Baldelomar E.J., Dowling J., Charlton J.R., Forbes M.S., Ng A. (2014). MRI-based glomerular morphology and pathology in whole human kidneys. Am. J. Physiol. Ren. Physiol..

[27] Hales C.N., Barker D.J. (2001). The thrifty phenotype hypothesis. Br. Med. Bull..

[28] Gluckman P.D., Hanson M.A. (2004). Living with the past: Evolution, development, and patterns of disease. Science.

[29] Cianfarani S., Germani D., Branca F. (1999). Low birthweight and adult insulin resistance: The “catch-up growth” hypothesis. Arch. Dis. Child. Fetal Neonatal..

[30] Pham T.D., MacLennan N.K., Chiu C.T., Laksana G.S., Hsu J.L., Lane R.H. (2003). Uteroplacental insufficiency increases apoptosis and alters *p53* gene methylation in the full-term IUGR rat kidney. Am. J. Physiol. Regul. Integr. Comp. Physiol..

[31] Lelièvre-Pégorier M., Vilar J., Ferrier M.L., Moreau E., Freund N., Gilbert T., Merlet-Bénichou C. (1998). Mild vitamin A deficiency leads to inborn nephron deficit in the rat. Kidney Int..

[32] Koleganova N., Piecha G., Ritz E., Becker L.E., Müller A., Weckbach M., Nyengaard J.R., Schirmacher P., Gross-Weissmann M.L. (2011). Both high and low maternal salt intake in pregnancy alter kidney development in the offspring. Am. J. Physiol. Ren. Physiol..

[33] Merlet-Bénichou C., Gilbert T., Muffat-Joly M., Lelièvre-Pégorier M., Leroy B. (1994). Intrauterine growth retardation leads to a permanent nephron deficit in the rat. Pediatr. Nephrol..

[34] Gray S.P., Denton K.M., Cullen-McEwen L., Bertram J.F., Moritz K.M. (2010). Prenatal exposure to alcohol reduces nephron number and raises blood pressure in progeny. J. Am. Soc. Nephrol..

[35] Hao X.Q., Zhang H.G., Yuan Z.B., Yang D.L., Hao L.Y., Li X.H. (2010). Prenatal exposure to lipopolysaccharide alters the intrarenal renin-angiotensin system and renal damage in offspring rats. Hypertens. Res..

[36] Celsi G., Kistner A., Aizman R., Eklöf A.C., Ceccatelli S., de Santiago A., Jacobson S.H. (1998). Prenatal dexamethasone causes oligonephronia, sodium retention, and higher blood pressure in the offspring. Pediatr. Res..

[37] Ortiz L.A., Quan A., Weinberg A., Baum M. (2001). Effect of prenatal dexamethasone on rat renal development. Kidney Int..

[38] Luzardo R., Silva P.A., Einicker-Lamas M., Ortiz-Costa S., do Carmo Mda G., Vieira-Filho L.D., Paixão A.D., Lara L.S., Vieyra A. (2011). Metabolic programming during lactation stimulates renal Na^+^ transport in the adult offspring due to an early impact on local angiotensin II pathways. PLoS ONE.

[39] Slabiak-Blaz N., Adamczak M., Gut N., Grajoszek A., Nyengaard J.R., Ritz E., Wiecek A. (2015). Administration of cyclosporine a in pregnant rats—The effect on blood pressure and on the glomerular number in their offspring. Kidney Blood Press. Res..

[40] Tain Y.L., Hsieh C.S., Lin I.C., Chen C.C., Sheen J.M., Huang L.T. (2010). Effects of maternal l-citrulline supplementation on renal function and blood pressure in offspring exposed to maternal caloric restriction: The impact of nitric oxide pathway. Nitric Oxide.

[41] Tain Y.L., Lee W.C., Hsu C.N., Lee W.C., Huang L.T., Lee C.T., Lin C.Y. (2013). Asymmetric dimethylarginine is associated with developmental programming of adult kidney disease and hypertension in offspring of streptozotocin-treated mothers. PLoS ONE.

[42] Paixão A.D., Maciel C.R., Teles M.B., Figueiredo-Silva J. (2001). Regional Brazilian diet-induced low birth weight is correlated with changes in renal hemodynamics and glomerular morphometry in adult age. Biol. Neonate.

[43] Woods L.L., Morgan T.K., Resko J.A. (2010). Castration fails to prevent prenatally programmed hypertension in male rats. Am. J. Physiol. Regul. Integr. Comp. Physiol..

[44] Tain Y.L., Chen C.C., Sheen J.M., Yu H.R., Tiao M.M., Kuo H.C., Huang L.T. (2014). Melatonin attenuates prenatal dexamethasone-induced blood pressure increase in a rat model. J. Am. Soc. Hypertens..

[45] Lisle S.J., Lewis R.M., Petry C.J., Ozanne S.E., Hales C.N., Forhead A.J. (2003). Effect of maternal iron restriction during pregnancy on renal morphology in the adult rat offspring. Br. J. Nutr..

[46] Hokke S., Puelles V.G., Armitage J.A., Fong K., Bertram J.F., Cullen-McEwen L.A. (2016). Maternal fat feeding augments offspring nephron endowment in mice. PLoS ONE.

[47] Woods L.L., Ingelfinger J.R., Rasch R. (2005). Modest maternal protein restriction fails to program adult hypertension in female rats. Am. J. Physiol. Regul. Integr. Comp. Physiol..

[48] Boubred F., Buffat C., Feuerstein J.M., Daniel L., Tsimaratos M., Oliver C., Lelièvre-Pégorier M., Simeoni U. (2007). Effects of early postnatal hypernutrition on nephron number and long-term renal function and structure in rats. Am. J. Physiol. Ren. Physiol..

[49] Thompson L.P., Al-Hasan Y. (2012). Impact of oxidative stress in fetal programming. J. Pregnancy.

[50] Tain Y.L., Leu S., Wu K.L., Lee W.C., Chan J.Y. (2014). Melatonin prevents maternal fructose intake-induced programmed hypertension in the offspring: Roles of nitric oxide and arachidonic acid metabolites. J. Pineal Res..

[51] Tai I.H., Sheen J.M., Lin Y.J., Yu H.R., Tiao M.M., Chen C.C., Huang L.T., Tain Y.L. (2016). Maternal *N*-acetylcysteine therapy regulates hydrogen sulfide-generating pathway and prevents programmed hypertension in male offspring exposed to prenatal dexamethasone and postnatal high-fat diet. Nitric Oxide.

[52] Tain Y.L., Hsu C.N., Lee C.T., Lin Y.J., Tsai C.C. (2016). *N*-Acetylcysteine prevents programmed hypertension in male rat offspring born to suramin-treated mothers. Biol. Reprod..

[53] Tain Y.L., Lee C.T., Chan J.Y., Hsu C.N. (2016). Maternal melatonin or *N*-acetylcysteine therapy regulates hydrogen sulfide-generating pathway and renal transcriptome to prevent prenatal *N*(G)-Nitro-l-arginine-methyl ester (l-NAME)-induced fetal programming of hypertension in adult male offspring. Am. J. Obstet. Gynecol..

[54] Stangenberg S., Nguyen L.T., Chen H., Al-Odat I., Killingsworth M.C., Gosnell M.E., Anwer A.G., Goldys E.M., Pollock C.A., Saad S. (2015). Oxidative stress, mitochondrial perturbations and fetal programming of renal disease induced by maternal smoking. Int. J. Biochem. Cell Biol..

[55] Cambonie G., Comte B., Yzydorczyk C., Ntimbane T., Germain N., Lê N.L., Pladys P., Gauthier C., Lahaie I., Abran D. (2007). Antenatal antioxidant prevents adult hypertension, vascular dysfunction, and microvascular rarefaction associated with in utero exposure to a low-protein diet. Am. J. Physiol. Regul. Integr. Comp. Physiol..

[56] Tain Y.L., Huang L.T. (2014). Restoration of asymmetric dimethylarginine-nitric oxide balance to prevent the development of hypertension. Int. J. Mol. Sci..

[57] Tain Y.L. (2015). Targeting redox balance to deprogramme obesity: Are we starting early enough?. J. Physiol..

[58] Yosypiv I.V. (2011). Renin-angiotensin system in ureteric bud branching morphogenesis: Insights into the mechanisms. Pediatr. Nephrol..

[59] Te Riet L., van Esch J.H., Roks A.J., van den Meiracker A.H., Danser A.H. (2015). Hypertension: Renin-angiotensin-aldosterone system alterations. Circ. Res..

[60] Bogdarina I., Welham S., King P.J., Burns S.P., Clark A.J. (2007). Epigenetic modification of the renin-angiotensin system in the fetal programming of hypertension. Circ. Res..

[61] Chappell M.C., Marshall A.C., Alzayadneh E.M., Shaltout H.A., Diz D.I. (2014). Update on the Angiotensin converting enzyme 2-Angiotensin (1–7)-MAS receptor axis: Fetal programing, sex differences, and intracellular pathways. Front. Endocrinol..

[62] Sherman R.C., Langley-Evans S.C. (1998). Early administration of angiotensin-converting enzyme inhibitor captopril, prevents the development of hypertension programmed by intrauterine exposure to a maternal low-protein diet in the rat. Clin. Sci..

[63] Sherman R.C., Langley-Evans S.C. (2000). Antihypertensive treatment in early postnatal life modulates prenatal dietary influences upon blood pressure in the rat. Clin. Sci..

[64] Manning J., Vehaskari V.M. (2005). Postnatal modulation of prenatally programmed hypertension by dietary Na and ACE inhibition. Am. J. Physiol. Regul. Integr. Comp. Physiol..

[65] Hsu C.N., Lee C.T., Huang L.T., Tain Y.L. (2015). Aliskiren in early postnatal life prevents hypertension and reduces asymmetric dimethylarginine in offspring exposed to maternal caloric restriction. J. Renin Angiotensin Aldosterone Syst..

[66] Hsu C.N., Wu K.L., Lee W.C., Leu S., Chan J.Y., Tain Y.L. (2016). Aliskiren administration during early postnatal life sex-specifically alleviates hypertension programmed by maternal high fructose consumption. Front. Physiol..

[67] Dagan A., Kwon H.M., Dwarakanath V., Baum M. (2008). Effect of renal denervation on prenatal programming of hypertension and renal tubular transporter abundance. Am. J. Physiol. Ren. Physiol..

[68] Tain Y.L., Lee W.C., Leu S., Wu K., Chan J. (2015). High salt exacerbates programmed hypertension in maternal fructose-fed male offspring. Nutr. Metab. Cardiovasc. Dis..

[69] Vallon V., Lang F. (2005). New insights into the role of serum- and glucocorticoid-inducible kinase SGK1 in the regulation of renal function and blood pressure. Curr. Opin. Nephrol. Hypertens..

[70] Rexhepaj R., Boini K.M., Huang D.Y., Amann K., Artunc F., Wang K., Brosens J.J., Kuhl D., Lang F. (2008). Role of maternal glucocorticoid inducible kinase SGK1 in fetal programming of blood pressure in response to prenatal diet. Am. J. Physiol. Regul. Integr. Comp. Physiol..

[71] Liu L., Barajas L. (1993). The rat renal nerves during development. Anat. Embryol..

[72] Barajas L., Liu L. (1993). The renal nerves in the newborn rat. Pediatr. Nephrol..

[73] Jansson T., Lambert G.W. (1999). Effect of intrauterine growth restriction on blood pressure, glucose tolerance and sympathetic nervous system activity in the rat at 3–4 months of age. J. Hypertens..

[74] Alexander B.T., Hendon A.E., Ferril G., Dwyer T.M. (2005). Renal denervation abolishes hypertension in low-birth-weight offspring from pregnant rats with reduced uterine perfusion. Hypertension.

[75] Sheen J.M., Yu H.R., Tiao M.M., Chen C.C., Huang L.T., Chang H.Y., Tain Y.L. (2015). Prenatal dexamethasone-induced programmed hypertension and renal programming. Life Sci..

[76] Tain Y.L., Wu K.L., Lee W.C., Leu S., Chan J.Y. (2015). Maternal fructose-intake-induced renal programming in adult male offspring. J. Nutr. Biochem..

[77] Moisiadis V.G., Matthews S.G. (2014). Glucocorticoids and fetal programming part 2: Mechanisms. Nat. Rev. Endocrinol..

[78] Langley-Evans S.C., Phillips G.J., Benediktsson R. (1996). Protein intake in pregnancy, placental glucocorticoid metabolism and the programming of hypertension in the rat. Placenta.

[79] Mairesse J., Lesage J., Breton C., Breant B., Hahn T., Darnaudery M., Dickson S.L., Seckl J., Blondeau B., Vieau D. (2007). Maternal stress alters endocrine function of the feto-placental unit in rats. Am. J. Physiol. Endocrinol. Metab..

[80] Kosicka K., Siemiątkowska A., Główka F.K. (2016). 11β-Hydroxysteroid dehydrogenase 2 in preeclampsia. Int. J. Endocrinol..

[81] Cottrell E.C., Seckl J.R. (2009). Prenatal stress, glucocorticoids and the programming of adult disease. Front. Behav. Neurosci..

[82] Singh R.R., Moritz K.M., Bertram J.F., Cullen-McEwen L.A. (2007). Effects of dexamethasone exposure on rat metanephric development: In vitro and in vivo studies. Am. J. Physiol. Ren. Physiol..

[83] Singh R.R., Cuffe J.S., Moritz K.M. (2012). Short- and long-term effects of exposure to natural and synthetic glucocorticoids during development. Clin. Exp. Pharmacol. Physiol..

[84] Marcotte M., Sharma R., Bouchard M. (2014). Gene regulatory network of renal primordium development. Pediatr. Nephrol..

[85] Cacalano G., Fariñas I., Wang L.C., Hagler K., Forgie A., Moore M., Armanini M., Phillips H., Ryan A.M., Reichardt L.F. (1998). GFRα1 is an essential receptor component for GDNF in the developing nervous system and kidney. Neuron.

[86] Bird A. (2007). Perceptions of epigenetics. Nature.

[87] Rees W.D., Hay S.M., Brown D.S., Antipatis C., Palmer R.M. (2000). Maternal protein deficiency causes hypermethylation of DNA in the livers of rat fetuses. J. Nutr..

[88] Suter M., Ma J., Harris A., Patterson L., Brown K.A., Shope C., Showalter L., Abramovici A., Aagaard-Tillery K.M. (2011). Maternal tobacco use modestly alters correlated epigenome-wide placental DNA methylation and gene expression. Epigenetics.

[89] Sable P., Randhir K., Kale A., Chavan-Gautam P., Joshi S. (2015). Maternal micronutrients and brain global methylation patterns in the offspring. Nutr. Neurosci..

[90] Ly A., Ishiguro L., Kim D., Im D., Kim S.E., Sohn K.J., Croxford R., Kim Y.I. (2016). Maternal folic acid supplementation modulates DNA methylation and gene expression in the rat offspring in a gestation period-dependent and organ-specific manner. J. Nutr. Biochem..

[91] Wu T.H., Kuo H.C., Lin I.C., Chien S.J., Huang L.T., Tain Y.L. (2014). Melatonin prevents neonatal dexamethasone induced programmed hypertension: Histone deacetylase inhibition. J. Steroid Biochem. Mol. Biol..

[92] Liu N., Zhuang S. (2015). Treatment of chronic kidney diseases with histone deacetylase inhibitors. Front. Physiol..

[93] Floris I., Kraft J.D., Altosaar I. (2016). Roles of microRNA across prenatal and postnatal periods. Int. J. Mol. Sci..

[94] Khorram O., Han G., Bagherpour R., Magee T.R., Desai M., Ross M.G., Chaudhri A.A., Toloubeydokhti T., Pearce W.J. (2010). Effect of maternal undernutrition on vascular expression of micro and messenger RNA in newborn and aging offspring. Am. J. Physiol. Regul. Integr. Comp. Physiol..

[95] Sene Lde B., Mesquita F.F., de Moraes L.N., Santos D.C., Carvalho R., Gontijo J.A., Boer P.A. (2013). Involvement of renal corpuscle microRNA expression on epithelial-to-mesenchymal transition in maternal low protein diet in adult programmed rats. PLoS ONE.

[96] Mouillet J.F., Chu T., Hubel C.A., Nelson D.M., Parks W.T., Sadovsky Y. (2010). The levels of hypoxia-regulated microRNAs in plasma of pregnant women with fetal growth restriction. Placenta.

[97] Tomat A.L., Salazar F.J. (2014). Mechanisms involved in developmental programming of hypertension and renal diseases. Gender differences. Horm. Mol. Biol. Clin. Investig..

[98] Ojeda N.B., Intapad S., Alexander B.T. (2014). Sex differences in the developmental programming of hypertension. Acta Physiol..

[99] Hilliard L.M., Sampson A.K., Brown R.D., Denton K.M. (2013). The “his and hers” of the renin-angiotensin system. Curr. Hypertens. Rep..

[100] Vina J., Gambini J., Lopez-Grueso R., Abdelaziz K.M., Jove M., Borras C. (2011). Females live longer than males: Role of oxidative stress. Curr. Pharm. Des..

[101] Kwekel J.C., Desai V.G., Moland C.L., Vijay V., Fuscoe J.C. (2013). Sex differences in kidney gene expression during the life cycle of F344 rats. Biol. Sex Differ..

[102] Tain Y.L., Wu M.S., Lin Y.J. (2016). Sex differences in renal transcriptome and programmed hypertension in offspring exposed to prenatal dexamethasone. Steroids.

[103] Mao J., Zhang X., Sieli P.T., Falduto M.T., Torres K.E., Rosenfeld C.S. (2010). Contrasting effects of different maternal diets on sexually dimorphic gene expression in the murine placenta. Proc. Natl. Acad. Sci. USA.

[104] Cox L.A., Li C., Glenn J.P., Lange K., Spradling K.D., Nathanielsz P.W., Jansson T. (2013). Expression of the placental transcriptome in maternal nutrient reduction in baboons is dependent on fetal sex. J. Nutr..

[105] Vaiman D., Gascoin-Lachambre G., Boubred F., Mondon F., Feuerstein J.M., Ligi I., Grandvuillemin I., Barbaux S., Ghigo E., Achard V. (2011). The intensity of IUGR-induced transcriptome deregulations is inversely correlated with the onset of organ function in a rat model. PLoS ONE.

[106] Buffat C., Boubred F., Mondon F., Chelbi S.T., Feuerstein J.M., Lelièvre-Pégorier M., Vaiman D., Simeoni U. (2007). Kidney gene expression analysis in a rat model of intrauterine growth restriction revealsmassive alterations of coagulation genes. Endocrinology.

[107] Almon R.R., Lai W., DuBois D.C., Jusko W.J. (2005). Corticosteroid-regulated genes in rat kidney: Mining time series array data. Am. J. Physiol. Endocrinol..

[108] Tain Y.L., Huang L.T., Chan J.Y., Lee C.T. (2015). Transcriptome analysis in rat kidneys: Importance of genes involved in programmed hypertension. Int. J. Mol. Sci..

[109] Tain Y.L., Hsu C.N., Chan J.Y., Huang L.T. (2015). Renal Transcriptome analysis of programmed hypertension induced by maternal nutritional insults. Int. J. Mol. Sci..

[110] Ruan X., Zheng F., Guan Y. (2008). PPARs and the kidney in metabolic syndrome. Am. J. Physiol. Ren. Physiol..

[111] Tain Y.L., Hsu C.N., Chan J. (2016). PPARs link early life nutritional insults to later programmed hypertension and metabolic syndrome. Int. J. Mol. Sci..

[112] Griffith O.W. (1999). Biologic and pharmacologic regulation of mammalian glutathione synthesis. Free Radic. Biol. Med..

[113] Tain Y.L., Lee W.C., Wu K.L., Leu S., Chan J.Y. (2016). Targeting arachidonic acid pathway to prevent programmed hypertension in maternal fructose-fed male adult rat offspring. J. Nutr. Biochem..

[114] Lu P.C., Sheen J.M., Yu H.R., Lin Y.J., Chen C.C., Tiao M.M., Tsai C.C., Huang L.T., Tain Y.L. (2016). Early postnatal treatment with soluble epoxide hydrolase inhibitor or 15-deoxy-Δ(12,14)-prostagandin J2 prevents prenatal dexamethasone and postnatal high saturated fat diet induced programmed hypertension in adult rat offspring. Prostaglandins Other Lipid Mediat..

[115] Costantini F. (2010). GDNF/Ret signaling and renal branching morphogenesis: From mesenchymal signals to epithelial cell behaviors. Organogenesis.

[116] Vieira-Filho L.D., Cabral E.V., Santos F.T., Coimbra T.M., Paixão A.D. (2011). α-Tocopherol prevents intrauterine undernutrition-induced oligonephronia in rats. Pediatr. Nephrol..

[117] Khodus G.R., Kruusmägi M., Li J., Liu X.L., Aperia A. (2011). Calcium signaling triggered by ouabain protects the embryonic kidney from adverse developmental programming. Pediatr. Nephrol..

[118] Makrakis J., Zimanyi M.A., Black M.J. (2007). Retinoic acid enhances nephron endowment in rats exposed to maternal protein restriction. Pediatr. Nephrol..

